# Implementation of a Program to Enhance Cognitive Reserve in Patients With Multiple Sclerosis (EM Reserva Program)

**DOI:** 10.31083/RN48330

**Published:** 2026-04-22

**Authors:** Miriam Ben-Yelun Insenser, Rocío Lopez Ruiz, Mónica Borges Guerra, Elvira Vázquez Domínguez, Eloisa Rubio Perez, Sara Madueño Eichau

**Affiliations:** ^1^Multiple Sclerosis Unit, Hospital Virgen Macarena, 41009 Sevilla, Spain; ^2^Fundación para la gestión de la Investigación en Salud de Sevilla, 41013 Sevilla, Spain

**Keywords:** cognitive reserve, multiple sclerosis, cognitive rehabilitation

## Abstract

**Background:**

Cognitive reserve (CR) may help mitigate cognitive decline in people with multiple sclerosis (PwMS); however, few interventions have targeted CR enhancement, and none have focused on individuals without baseline cognitive impairment. The EM Reserva program is a multimodal intervention that combines cognitive leisure activities, aerobic exercise, and structured social engagement and is designed to strengthen CR in individuals with relapsing-remitting MS (RRMS).

**Methods:**

This pragmatic, single-center, observer-blinded randomized controlled trial included PwMS aged 18–55 years with RRMS and no cognitive impairment. Participants were randomized in a 1:1 ratio to either the EM Reserva program or usual cognitive care. Outcomes were assessed at baseline and at 6-month and 12-month time points. The primary endpoint was the change in Symbol Digit Modalities Test (SDMT) scores at 6 months. Secondary outcomes included additional neuropsychological measures, Modified Fatigue Impact Scale-5 (MFIS-5), Perceived Deficits Questionnaire-5 (PDQ-5), and Multiple Sclerosis Quality of Life‑54 (MSQOL-54). Analyses followed a modified intention-to-treat approach.

**Results:**

Forty-five participants completed follow-up. At 6 months, the EM Reserva group showed a significant improvement in SDMT scores compared with controls (mean difference –4.23, *p* < 0.022) and higher Controlled Oral Word Association Test (COWAT) scores. These cognitive gains were not sustained at 12 months. Fatigue improved in both groups at 6 months but remained significantly lower only in the EM Reserva group at 12 months. No between-group differences were observed in PDQ-5, MSQOL-54, or other neuropsychological measures.

**Conclusion:**

The EM Reserva program produced short-term improvements in processing speed and verbal fluency in cognitively preserved PwMS, along with sustained reductions in fatigue. However, cognitive benefits were not maintained at 12 months, and subjective cognitive functioning remained unchanged. These findings suggest that multimodal CR-oriented interventions may offer temporary cognitive advantages, but long-term maintenance strategies are likely required to sustain gains.

**Clinical Trial Registration:**

No: NCT05546424. https://clinicaltrials.gov/study/NCT05546424.

## 1. Introduction

Multiple sclerosis (MS) is a chronic, inflammatory, and degenerative disease that affects the central nervous system, causing cognitive impairment in approximately 40–60% of patients [1, 2, 3]. The cognitive profile in people with MS (PwMS) is generally characterized by impairment in information processing speed, attention, memory, and executive functions [4, 5, 6].

Although the impact of cognitive impairment on the daily lives of people with MS varies greatly [7, 8], generally, PwMS and cognitive dysfunction face greater difficulties finding employment, engaging in social activities, requiring more personal assistance, and have a lower quality of life [6, 7, 8, 9].

Cognitive reserve (CR) is defined as the adaptability that helps to explain differential susceptibility of cognitive abilities or day-to-day function to brain aging, pathology, or insult [10], and may have a potential role in explaining the variability between structural brain damage, cognitive impairment, and its impact in PwMS [11, 12]. CR is the result of the cumulative effect of lifelong intellectual enrichment, including educational and occupational achievements, lexical abilities, and cognitive leisure activities [11, 12, 13].

CR has been studied extensively in relation to Alzheimer’s Disease. Various studies have shown that individuals who engage in cognitively stimulating activities have a lower risk of developing this disease [14, 15]. In general, a high CR is associated with preserved global cognitive function even in the presence of brain pathologies [16]. Regarding MS, some studies highlighted that a high CR can alleviate the severity and delay the appearance of cognitive impairment in PwMS [17, 18].

CR is understood to be a dynamic process that can be enhanced through intellectually enriching activities at any point in life. To date, there are few intervention studies on enhancing cognitive reserve in PwMS. Recently, a study demonstrated that patients who underwent a comprehensive cognitive rehabilitation program showed improved cognitive outcomes compared with a group that received only instructions to perform physical exercise [19]. However, to date, the impact of cognitive reserve enhancement in PwMS without cognitive impairment has never been evaluated.

According to previous literature [12], three activities strongly enhance CR: cognitive leisure tasks, aerobic physical exercise, and activities that promote social relationships. Cognitive leisure tasks include reading, producing art, writing, playing musical instruments, playing structured games, and engaging in hobbies.

For these reasons, we propose to evaluate the impact of the “EM Reserva” study, a specific cognitive exercise program designed to enhance cognitive reserve, and to assess its effectiveness in improving or maintaining the cognitive profile of individuals with multiple sclerosis who do not have baseline cognitive impairment. This study will compare the effects of the EM Reserva cognitive training program with the standard cognitive exercise recommendations provided by expert neuropsychologists in a multiple sclerosis unit. Additionally, the study will assess the impact of the EM Reserva program on mood and quality of life compared to the usual cognitive exercise guidelines.

## 2. Objectives 

### 2.1 Primary Objective

To evaluate the efficacy of the EM Reserva intervention in improving short-term information processing speed.

### 2.2 Primary Endpoint

Change in Symbol Digit Modalities Test (SDMT) mean scores at 6 months compared with baseline.

### 2.3 Secondary Objectives

1. To evaluate mid-term changes in information processing speed.

2. To compare the effects of EM Reserva versus usual cognitive care on memory, attention, and executive functions at 6 and 12 months.

3. To assess the impact of EM Reserva on quality of life and disease-related symptoms at 6 and 12 months.

### 2.4 Secondary Endpoints

1. Persistence of SDMT changes at 12 months.

2. Changes in Trail Making Test A–B (TMT A–B), Brief Neuropsychological Battery of Rao (BRB-N), Wechsler Abbreviated Intelligence Scale IV (WAIS-IV), Tower of London, and phonemic/semantic fluency scores at 6 and 12 months.

3. Changes in Multiple Sclerosis Quality of Life‑54 (MSQOL-54), perceived cognitive deficits (PDQ-5), Modified Fatigue Impact Scale-5 (MFIS-5), and Beck Depression Inventory (BDI) scores at 6 and 12 months.

## 3. Materials and Methods

This pragmatic, single-center, observer-blinded randomized controlled trial (RCT) compared the EM Reserva cognitive program with usual cognitive care among PwMS without cognitive impairment. The study was conducted at the Multiple Sclerosis Unit of Virgen Macarena University Hospital (UEMAC), Seville, Spain.

### 3.1 Participants

PwMS with relapsing-remitting MS (RRMS) under regular follow-up at UEMAC were screened. Cognitive impairment was defined according to Amato’s criteria: failure on at least two BRB-N tests, with scores ≥1.5 standard deviation (SD) below normative values.

### 3.2 Inclusion Criteria

∙ Aged 18–55 years (inclusive).

∙ Diagnosed with RRMS according to the 2017 McDonald Revised Criteria [20].

∙ Mild to moderate physical impairment (Expanded Disability Status Scale (EDSS) <3).

∙ Less than 15 years since disease onset.

∙ No cognitive impairment as assessed by the BRB-N (Form A), corrected for age and education, and performed within the last three months according to Amato’s criteria.

∙ Ability to attend group sessions.

∙ Ability to provide written informed consent.

### 3.3 Exclusion Criteria

∙ Diagnosis of secondary progressive or primary progressive MS according to the 2017 revised McDonald criteria [20].

∙ Relapse or corticosteroid treatment in the three months before study inclusion.

∙ Vision or hearing problems that prevent the completion of cognitive assessments.

∙ Severe medical or psychiatric conditions that prevent engagement in treatment, or a Beck Depression Inventory score >20.

∙ Participation in other psychological intervention programs.

Neurologists collected demographic and clinical data. Neuropsychologists performed cognitive assessments and administered the Cognitive Reserve Questionnaire (CRQ) [21].

### 3.4 Instruments and Measurements

To screen for cognitive impairment in individuals with multiple sclerosis, the validated BRB-N scale is used. Participants who meet all inclusion criteria, do not meet any exclusion criteria, and consent to participate in the study undergo an extended cognitive assessment. This includes:

∙ The TMT A–B is used to evaluate prefrontal functions.

∙ The Wechsler Abbreviated Intelligence Scale IV (WAIS-IV) is used to assess executive functions and working memory.

∙ The Tower of London test is used to evaluate planning, cognitive flexibility, and inhibitory control.

∙ The Controlled Oral Word Association Test (COWAT) and the WAIS are used to assess lexical-semantic recall and estimate vocabulary.

The CRQ is also administered [21]. This questionnaire consists of eight items that measure various aspects of the participant’s intellectual activity considered important in the formation of cognitive reserve, such as education, training courses, parental education, and occupation. A CRQ score ≤6 places the subject’s cognitive reserve in the lower range. Scores between 7 and 9 indicate a medium-low range of cognitive reserve, while scores between 10 and 14 indicate a medium-high range. Scores of ≥15 points are classified as a high level of cognitive reserve.

Finally, quality of life is assessed using the MSQOL-54 test. The PDQ-5 is used to evaluate the patient’s perceived impact of cognitive impairments, fatigue is measured with the MFIS-5 scale, and mood is evaluated using the BDI.

### 3.5 Intervention Protocol

Fifty-eight patients with RRMS and without cognitive impairment (CI) are assigned to either the “EM Reserva” intervention group or the usual cognitive care group in a 1:1 ratio.

The sample size was determined by calculating the number of individuals required to achieve the study’s primary objective, informed by results from previous studies on MS patients [22].

Randomization is stratified by Cognitive Reserve Questionnaire scores and age and is conducted via an Internet-based system to ensure impartial allocation.

### 3.6 Control Group

Participants in the control group receive general cognitive exercises and advice from MS neuropsychologists. They are instructed to:

∙ Complete daily cognitive exercises (such as sudokus, crosswords, and mazes) for 30 minutes.

∙ Read newspapers or magazines for an additional 15 minutes each day.

### 3.7 “EM Reserva” Intervention Group

Participants in the “EM Reserva” intervention group engage in a comprehensive cognitive enhancement program that includes:

1. Cognitive Leisure Tasks:

∘ Daily reading of books and magazines.

∘ Creative tasks such as drawing, writing, or playing a musical instrument.

2. Physical Exercise:

∘ Group workouts were conducted via a virtual platform for 1 hour, 3 times weekly, and were guided by a neuro physiotherapist.

3. Promoting Social Relationships:

∘ Group piano lessons.

∘ Board games.

∘ Cultural activities such as museum visits.

Participants in the “EM Reserva” group are required to perform at least 30 minutes of individual cognitive exercises at home daily (including musical exercises with piano, reading, and writing), engage in physical exercise three times weekly for one hour, and participate in social activities in groups of six participants, meeting for one hour weekly for five months.

Attendance and compliance are monitored, with catch-up sessions available for any missed content. Outcome assessments are conducted at baseline, 6, and 12 months.

Additional data collected by specialized MS neurologists, blinded to the intervention, include EDSS scores, number of relapses, number of new/enlarged T2 lesions on cerebral magnetic resonance imaging (MRI), and concomitant medications.

### 3.8 Statistical Analysis 

Quantitative variables were summarized using measures of central tendency and dispersion, and qualitative variables using absolute and relative frequencies. Outcomes were analyzed using a repeated-measures model including group, time, and a group×time interaction, and adjusted marginal means were used to estimate between-group differences. Differences between groups are reported with their corresponding 95% confidence intervals. The primary endpoint was the change in SDMT scores at 6 months. The modified intention-to-treat (mITT) population included all randomized participants with available outcome data at the relevant time points and was analyzed according to their randomized group.

A *p*-value < 0.05 was considered statistically significant. No correction for multiple comparisons was applied. Sample size calculations indicated that 29 participants per group were required to achieve 80% power at α < 0.05, based on estimates obtained using STATA v15.1 (StataCorp LLC, College Station, TX, USA). Generalized Linear Models were used to estimate marginal means for each outcome. All statistical analyses were conducted using R version 4.3.2 (R Core Team, 2023; R Foundation for Statistical Computing, Vienna, Austria), SPSS v29 (IBM Corp., Armonk, NY, USA), and STATA v15.1 (StataCorp LLC, College Station, TX, USA).

## 4. Results

A total of 58 participants met the inclusion criteria and were initially selected between May and November 2022. Considering the 12‑month follow‑up, the study was conducted between May 2022 and November 2023. Ten individuals did not initiate the intervention due to scheduling incompatibilities, leaving 48 participants who were randomized in a 1:1 ratio to the EM Reserva group or the control group. During follow-up, three additional participants were lost to follow-up, resulting in a final analysed sample of 45 participants. This attrition (22%) reduced the final sample size below the originally calculated 29 participants per group, which is acknowledged as a limitation regarding statistical power, particularly for long-term outcomes.

Baseline demographic and clinical characteristics were comparable between groups (Table 1). Participants were evaluated at baseline (V1), 6 months (V2), and 12 months (V3). Analyses followed a modified intention-to-treat approach, including all randomized participants who provided outcome data at follow-up. The data were processed between January 2024 and May 2024. The summary of the results is shown in Table 2. Data are presented as adjusted marginal means with standard errors in parentheses: Mean. Table 3 summarizes adjusted marginal means and between-group differences with 95% confidence intervals for the primary and secondary outcomes.

**Table 1.** **Demographic and clinical characteristics**.

		EM Reserva group A	Control group B
		n = 29	n = 29
Age (mean)		39.28	36.41
Sex			
	Male	6	2
	Female	23	27
Highest level of education completed			
	Primary	1	1
	Secondary/high school	10	8
	College/university	18	20
EDSS (mean)		1.5	1.25
Duration of MS (mean)		5	7
T2 lesions		<10: 16	<10: 11
		11–49: 12	11–49: 13
		>50: 1	>50: 5

EDSS, Expanded Disability Status Scale; MS, multiple sclerosis.

**Table 2.** **Summary results**.

	A: EM Reserva B: Control		Mean (SE)		
SDMT	IG (A)	Baseline (V1)	56.1 (2.75)		
		6 m (V2)	63.5 (2.75)	*p * < 0.022	
		12 m (V3)	56.5 (1.81)	*p * < 1	*p * < 0.298
	C (B)	Baseline (V1)	54.4 (2.35)		
		6 m (V2)	55.5 (2.35)		
		12 m (V3)	54.8 (2.35)		
MSQOL-54	IG (A)	Baseline (V1)	175 (4.02)		
		6 m (V2)	170 (3.90)		
		12 m (V3)	170 (3.90)		
	C (B)	Baseline (V1)	167 (3.28)		
		6 m (V2)	169 (3.31)		
		12 m (V3)	165 (3.25)		
MFIS-5	IG (A)	Baseline (V1)	7.65 (1.74)	*p * < 0.01	
		6 m (V2)	4.55 (1.24)	*p * < 0.01	
		12 m (V3)	3.64 (1.11)	*p * < 0.01	
	C (B)	Baseline (V1)	10.30 (1.83)	*p * < 0.01	
		6 m (V2)	4.27 (1.04)	*p * < 0.01	
		12 m (V3)	5.19 (1.15)		
PDQ-5	IG (A)	Baseline (V1)	3.73 (0.74)		
		6 m (V2)	4.34 (0.89)		
		12 m (V3)	4.23 (0.88)		
	C (B)	Baseline (V1)	6.35 (1.11)		
		6 m (V2)	3.99 (0.70)	*p * < 0.09	
		12 m (V3)	5.46 (0.96)		

SE, standard error; IG, intervention group; C, control group; SDMT, Symbol Digit Modalities Test; MSQOL‑54, Multiple Sclerosis Quality of Life‑54; MFIS, Modified Fatigue Impact Scale; PDQ, Perceived Deficits Questionnaire.

**Table 3.** **Adjusted marginal means and between‑group differences (95% CI)**.

Outcome	Timepoint	EM Reserva Mean (SE)	Control Mean (SE)	Difference (EM-R Control)	95% CI	*p*-value
SDMT	6 months	63.5 (2.75)	55.5 (2.35)	−4.23	−7.25 to −1.21	0.022
SDMT	12 months	56.5 (1.81)	54.8 (2.35)	−1.19	−4.21 to 1.82	0.298
COWAT‑Animals	6 months	27.4 (1.17)	23.6 (1.00)	2.94	0.37 to 5.51	0.030
COWAT-P	6 months	21.4 (1.23)	19.2 (1.05)	1.14	−1.53 to 3.81	0.407
MFIS-5	12 months	3.64 (1.11)	5.19 (1.15)	−1.55	−3.76 to 0.66	0.090

COWAT, Controlled Oral Word Association Test; COWAT-P, COWAT phonemic fluency.

### 4.1 SDMT at 6 and 12 Months

At baseline, mean SDMT scores were similar between groups. At 6 months, the EM Reserva group showed a statistically significant improvement, reaching a mean score of 63.5, whereas the control group showed minimal change. The between-group difference at 6 months was –4.23 points (standard error [SE] 1.54, 95% CI –7.25 to –1.21, *p* = 0.022), meeting the primary endpoint. At 12 months, SDMT scores in the EM Reserva group declined toward baseline levels, and no significant differences were observed between groups (difference –1.19 points, SE 1.54, 95% CI –4.21 to 1.82, *p* = 0.298). The attenuation of the effect may reflect the end of the structured intervention, reduced statistical power due to attrition, or practice-related effects.

### 4.2 Controlled Oral Word Association Test (COWAT)

Phonemic and semantic fluency improved temporarily in the EM Reserva group.

For semantic fluency (COWAT-Animals), adjusted marginal means were comparable at baseline (EM Reserva 24.7 [SE 1.17] vs. control 22.2 [SE 1.00]). At 6 months, the EM Reserva group reached 27.4 (SE 1.17), compared with 23.6 (SE 1.00) in the control group. The adjusted between-group difference at this time point was 2.94 points (SE 1.31, 95% CI 0.37 to 5.51, *p* = 0.030), indicating a significant short-term improvement. At 12 months, scores declined toward baseline levels, and no significant differences were observed between groups.

For phonemic fluency (COWAT-P), adjusted marginal means were similar at baseline (EM Reserva 19.7 [SE 1.23] vs. control 18.0 [SE 1.05]). At 6 months, scores increased to 21.4 (SE 1.23) in the EM Reserva group and to 19.2 (SE 1.05) in the control group. The adjusted between-group difference at this time point was 1.14 points (SE 1.36, 95% CI –1.53 to 3.81, *p* = 0.407), indicating no significant group effect. Scores returned to baseline at 12 months in both groups, with no significant differences across time points in pairwise comparisons.

### 4.3 Modified Fatigue Scale 5 Fatigue (MFIS-5)

Both groups showed reductions in fatigue scores at 6 months, but the improvement was more pronounced and sustained in the EM Reserva group. At 12 months, the between-group difference was –1.55 points (95% CI –3.76 to 0.66), indicating a favorable but non-significant trend. No linear association was found between specific program activities and MFIS-5 scores, although adjusted pairwise comparisons indicated significant improvements across time points.

### 4.4 Perceived Cognitive Deficits (PDQ-5)

No significant differences were observed between groups in PDQ-5 scores at any time point. This suggests that, despite objective improvements in processing speed at 6 months, participants did not perceive meaningful changes in their everyday cognitive functioning.

### 4.5 Changes in Other Tests

No significant differences between groups were found in additional cognitive tests (TMT A-B, BRB-N, WAIS-IV, Tower of London) or in MSQOL-54. Depression and anxiety scores also remained comparable between groups.

## 5. Discussion 

This study evaluated the impact of the EM Reserva program a multimodal cognitive reserve enhancement intervention on cognitive performance, fatigue, and quality of life in individuals with RRMS who had no baseline cognitive impairment. The program produced short-term improvements in processing speed and verbal fluency, as reflected by SDMT and COWAT scores at 6 months. However, these cognitive gains were not sustained at the 12-month follow-up. Several factors may explain the transient nature of the cognitive effects. Although the initial sample size calculation required 29 participants per group, only 45 participants completed follow-up assessments. This 22% attrition rate reduced the statistical power of the study, particularly for long-term outcomes. Therefore, the absence of significant differences at 12 months—especially for the primary endpoint—should be interpreted with caution, as a Type II error cannot be ruled out. This limitation is especially relevant given the modest effect sizes typically observed in cognitive rehabilitation studies in MS [23]. A meta-analysis from 2018 indicated that cognitive-focused interventions have a small effect on cognitive domains, along with minimal impact on measures of depression and anxiety [23]. Our findings align with previous studies, demonstrating positive effects of cognitive-focused interventions on working memory.

A major methodological consideration is the substantial difference in intervention intensity between groups. The EM Reserva program involved daily cognitive tasks, three supervised exercise sessions per week, and weekly structured social activities. In contrast, the control group received only brief recommendations for unsupervised cognitive exercises. This discrepancy may have introduced a Hawthorne effect, whereby improvements in the intervention group reflect increased attention, structure, and social engagement rather than the program’s specific cognitive content. Future trials should consider designing control conditions with comparable time and attention demands to minimize this bias.

The EM Reserva program integrates cognitive training, aerobic exercise, and social activities, consistent with the theoretical framework of cognitive reserve [11, 12, 13]. However, this multimodal design complicates attributing observed effects to specific components. For example, the sustained improvement in fatigue may be largely attributable to the aerobic exercise component, as physical activity is known to reduce fatigue in PwMS. Previous studies combining cognitive rehabilitation with aerobic exercise have shown mixed results regarding processing speed [24], and the present findings align with this complexity. Although our sample demonstrated short-term improvements in SDMT, it remains unclear whether these gains reflect cognitive training, physical exercise, social engagement, or a synergistic combination of these factors.

One of the most notable findings is the persistence of fatigue improvement at 12 months, despite the cessation of structured intervention at 6 months. This raises the possibility that participants may have maintained some of the exercise or cognitive habits acquired during the program. However, lifestyle behaviours between months 6 and 12 were not systematically monitored, limiting the ability to draw firm conclusions. Future studies should incorporate longer follow-up assessments of physical activity and cognitive engagement to better understand the mechanisms underlying sustained fatigue benefits.

The SDMT is known to exhibit practice effects, particularly in cognitively preserved individuals. The significant improvement observed at 6 months may reflect increased familiarity with the test rather than true neurocognitive enhancement. The subsequent decline at 12 months, once the structured intervention ended, is consistent with this interpretation. This limitation should be considered when interpreting short-term cognitive gains in populations without baseline impairment.

Participants in this study did not exhibit cognitive impairment at baseline, with mean SDMT scores already within the normal range. Although the 6-month improvement was statistically significant, the absence of corresponding changes in PDQ-5 suggests limited clinical relevance. This divergence between objective and subjective measures has been reported in previous cognitive rehabilitation studies. It highlights the challenge of demonstrating meaningful cognitive benefits in individuals who are already functioning well.

Previous studies combining cognitive rehabilitation with aerobic exercise have not consistently demonstrated improvements in processing speed [24]. For example, the CogEx trial, which included 311 individuals with more advanced progressive MS, did not show significant gains in SDMT performance despite a combined cognitive-exercise intervention. In contrast, our sample exhibited a short-term improvement in processing speed at 6 months. However, given the multimodal nature of the EM Reserva program, it remains difficult to determine the extent to which this effect is attributable to the exercise component, the cognitive training, or their interaction. This limitation underscores the need for future studies designed to isolate the contribution of each intervention element.

Recent research has further explored the efficacy of cognitive rehabilitation in MS. Notably, Jiménez-Morales *et al*. [19] reported that the Integrated Program of Cognitive Rehabilitation improved verbal and visual working memory and processing speed [25]. Similar to our approach, their intervention combined cognitive and physical components and was associated with an increase in the cognitive reserve index. These findings reinforce the potential value of targeting cognitive reserve as a therapeutic objective and suggest that multimodal interventions may offer meaningful benefits for selected MS populations.

## 6. Limitations

This study has several limitations. Attrition reduced the final sample size, limiting statistical power for long-term outcomes. The multimodal nature of the intervention prevents attribution of effects to specific components. The imbalance in intervention intensity between groups may have introduced a Hawthorne effect. Practice effects may have influenced SDMT performance. Finally, lifestyle behaviours after the intervention period were not monitored, which limits the interpretation of sustained improvements in fatigue.

These findings have several implications for clinical practice and future research. The transient nature of the cognitive improvements observed in this study highlights the need for cognitive programs that incorporate follow-up or maintenance components to sustain long-term benefits. Future interventions should explore strategies to promote continued engagement—such as technology-supported platforms or structured home-based activities—to reduce the decline in cognitive gains once supervised training ends. The sustained reduction in fatigue suggests that multimodal interventions may exert beneficial effects on non-cognitive symptoms; however, the specific mechanisms underlying this improvement remain unclear. Although no linear association between program activities and fatigue reduction was demonstrated, adjusted analyses indicated that additional cognitive habits, such as regular reading, may contribute to enhanced outcomes. Further research is needed to clarify how daily lifestyle behaviours and external cognitive stimulation interact with structured rehabilitation programs, and to determine which components are most effective for long-term symptom management.

## 7. Conclusion

The EM Reserva program produced short-term improvements in processing speed and verbal fluency in PwMS without cognitive impairment, along with sustained reductions in fatigue. However, these cognitive benefits were not maintained at 12-month follow-up, and no changes were observed in perceived cognitive functioning. These findings suggest that while multimodal cognitive reserve interventions may offer short-term cognitive advantages, long-term maintenance strategies are likely required. Future studies should incorporate active control groups, larger sample sizes, and extended follow-up monitoring to clarify the durability and clinical relevance of these effects.

## Availability of Data and Materials

All data supporting the findings of this study are available from the corresponding author upon reasonable request.

## References

[1] Amato MP, Zipoli V, Portaccio E (2006). Multiple sclerosis-related cognitive changes: a review of cross-sectional and longitudinal studies. *Journal of the Neurological Sciences*.

[2] Rao SM, Leo GJ, Bernardin L, Unverzagt F (1991). Cognitive dysfunction in multiple sclerosis. I. Frequency, patterns, and prediction. *Neurology*.

[3] Benedict RH, Cox D, Thompson LL, Foley F, Weinstock-Guttman B, Munschauer F (2004). Reliable screening for neuropsychological impairment in multiple sclerosis. *Multiple Sclerosis Journal*.

[4] Heaton RK, Nelson LM, Thompson DS, Burks JS, Franklin GM (1985). Neuropsychological findings in relapsing-remitting and chronic-progressive multiple sclerosis. *Journal of Consulting and Clinical Psychology*.

[5] Potagas C, Giogkaraki E, Koutsis G, Mandellos D, Tsirempolou E, Sfagos C (2008). Cognitive impairment in different MS subtypes and clinically isolated syndromes. *Journal of the Neurological Sciences*.

[6] Chiaravalloti ND, DeLuca J (2008). Cognitive impairment in multiple sclerosis. *The Lancet. Neurology*.

[7] Rao SM, Leo GJ, Ellington L, Nauertz T, Bernardin L, Unverzagt F (1991). Cognitive dysfunction in multiple sclerosis. II. Impact on employment and social functioning. *Neurology*.

[8] Amato MP, Prestipino E, Bellinvia A, Niccolai C, Razzolini L, Pastò L (2019). Cognitive impairment in multiple sclerosis: An exploratory analysis of environmental and lifestyle risk factors. *PloS One*.

[9] Achiron A, Barak Y (2003). Cognitive impairment in probable multiple sclerosis. *Journal of Neurology, Neurosurgery, and Psychiatry*.

[10] Stern Y, Arenaza-Urquijo EM, Bartrés-Faz D, Belleville S, Cantilon M, Chetelat G (2020). Whitepaper: Defining and investigating cognitive reserve, brain reserve, and brain maintenance. Alzheimer’s & Dementia: the Journal of the Alzheimer’s Association. *Alzheimer’s & Dementia: the Journal of the Alzheimer’s Association*.

[11] Sumowski JF (2015). Cognitive Reserve as a Useful Concept for Early Intervention Research in Multiple Sclerosis. *Frontiers in Neurology*.

[12] Sumowski JF, Leavitt VM (2013). Cognitive reserve in multiple sclerosis. *Multiple Sclerosis (Houndmills, Basingstoke, England)*.

[13] Nunnari D, De Cola MC, Costa A, Rifici C, Bramanti P, Marino S (2016). Exploring cognitive reserve in multiple sclerosis: New findings from a cross-sectional study. *Journal of Clinical and Experimental Neuropsychology*.

[14] Wilson RS, Mendes De Leon CF, Barnes LL, Schneider JA, Bienias JL, Evans DA (2002). Participation in cognitively stimulating activities and risk of incident Alzheimer disease. *JAMA*.

[15] Wilson RS, Bennett DA, Bienias JL, Aggarwal NT, Mendes De Leon CF, Morris MC (2002). Cognitive activity and incident AD in a population-based sample of older persons. *Neurology*.

[16] Li X, Song R, Qi X, Xu H, Yang W, Kivipelto M (2021). Influence of Cognitive Reserve on Cognitive Trajectories: Role of Brain Pathologies. *Neurology*.

[17] Sumowski JF, Rocca MA, Leavitt VM, Riccitelli G, Comi G, DeLuca J (2013). Brain reserve and cognitive reserve in multiple sclerosis: what you’ve got and how you use it. *Neurology*.

[18] Sumowski JF, Wylie GR, Deluca J, Chiaravalloti N (2010). Intellectual enrichment is linked to cerebral efficiency in multiple sclerosis: functional magnetic resonance imaging evidence for cognitive reserve. *Brain: a Journal of Neurology*.

[19] Jiménez-Morales RM, Broche-Pérez Y, Macías-Delgado Y, Sebrango C, Díaz-Díaz S, Castiñeira-Rodriguez R (2024). Cognitive rehabilitation program in patients with multiple sclerosis: A pilot study. *Neurologia*.

[20] Thompson AJ, Banwell BL, Barkhof F, Carroll WM, Coetzee T, Comi G (2018). Diagnosis of multiple sclerosis: 2017 revisions of the McDonald criteria. *The Lancet. Neurology*.

[21] Rami L, Valls-Pedret C, Bartrés-Faz D, Caprile C, Solé-Padullés C, Castellvi M (2011). Cognitive reserve questionnaire. Scores obtained in a healthy elderly population and in one with Alzheimer’s disease. *Revista De Neurologia*.

[22] Mani A, Chohedri E, Ravanfar P, Mowla A, Nikseresht A (2018). Efficacy of group cognitive rehabilitation therapy in multiple sclerosis. *Acta Neurologica Scandinavica*.

[23] Gromisch ES, Fiszdon JM, Kurtz MM (2020). The effects of cognitive-focused interventions on cognition and psychological well-being in persons with multiple sclerosis: A meta-analysis. *Neuropsychological Rehabilitation*.

[24] Feinstein A, Amato MP, Brichetto G, Chataway J, Chiaravalloti ND, Cutter G (2023). Cognitive rehabilitation and aerobic exercise for cognitive impairment in people with progressive multiple sclerosis (CogEx): a randomised, blinded, sham-controlled trial. *The Lancet. Neurology*.

[25] Lincoln NB, Bradshaw LE, Constantinescu CS, Day F, Drummond AE, Fitzsimmons D (2020). Cognitive rehabilitation for attention and memory in people with multiple sclerosis: a randomized controlled trial (CRAMMS). *Clinical Rehabilitation*.

